# Fast and Reliable On-Site Quality Assessment of Essential Raw Brewing Materials Using MicroNIR and Chemometrics

**DOI:** 10.3390/foods13172728

**Published:** 2024-08-28

**Authors:** Giuseppina Gullifa, Chiara Albertini, Elena Papa, Rita Petrucci, Paola Di Matteo, Martina Bortolami, Stefano Materazzi, Roberta Risoluti

**Affiliations:** 1 Department of Chemistry, Sapienza University of Rome, Piazzale Aldo Moro 5, 00185 Rome, Italy; giuseppina.gullifa@uniroma1.it (G.G.); chiara.albertini@uniroma1.it (C.A.); elena.papa@uniroma1.it (E.P.); stefano.materazzi@uniroma1.it (S.M.); 2 Department of Basic and Applied Sciences for Engineering, Sapienza University of Rome, Via Castro Laurenziano, 7, 00161 Roma, Italy; rita.petrucci@uniroma1.it (R.P.); p.dimatteo@uniroma1.it (P.D.M.); martina.bortolami@uniroma1.it (M.B.)

**Keywords:** microNIR, chemometrics, malts, innovative platform, quality control

## Abstract

The interest in the quality control of the raw materials, intermediates, and final products, as well as production methods, of beer has increased significantly in recent decades due to the needs and expectations of consumers. Increasing in the industrialization and globalization of beer supply chains led to a need for novel analytical tools suitable for the rapid and reliable characterization of the materials involved. In this study, an ultracompact instrument operating in the NIR region of the spectrum, microNIR, was tested for the chemical investigation of barley malts. The essential raw materials for brewing require careful control since they deeply affect the characteristic flavor and taste of the final products. Therefore, a robust prediction model able to classify base and specialty barley malts was developed starting from NIR measurements. Soft Independent Class Analogy (SIMCA) was selected as the chemometric technique for the optimization of two prediction models, and ground and sieved materials were investigated using spectroscopy. The microNIR/chemometric approach proposed in this study permitted the correct prediction of the malt samples included in the external validation set, providing false positive and false negative rates no higher than 3.41% and 0.25%, respectively, and confirming the feasibility of the novel analytical platform.

## 1. Introduction

Beer is the second most consumed alcoholic beverage worldwide so the market offers a wide range of beer products that differ in appearance (color, clarity, foam), flavor, and taste. These features play an important role in the quality of the resulting fermented beer, defining and reflecting the properties of the raw materials as well as the effects of the malting and brewing processes [1].

Beer is obtained with four basic starting ingredients: malt, water, yeast and hops. Barley is the most common raw cereal used for the production of malt, well known as barley malt; however, wheat, sorghum, and rye malts are also available. Steeping, germination, kilning, and roasting are the main stages of malting. On the basis of the conditions (moisture, temperature, airflow, and pH) set up for each stage, malts with different chemical compositions can be obtained [2,3,4,5]. Complex reactions, such as the Millard reaction, Strecker degradation, caramelization, lipid peroxidation, and degradation, promote the release of alcohols, aldehydes, ketones, organic acids, and lipids, which contribute to the malt’s flavor and thus the taste of the beer [6,7]. Two main classes of malts are recognized, base and specialty malts. Base malts are product via the standard protocol and can be used at 100% of the recipe, providing the traditional color and flavor of the final product. Specialty malts are responsible for sought-after colors and aromas such as toasted, fruity, and caramel flavors [6]. A careful analytical evaluation of these essential raw materials is increasingly required by brewers in order to ensure desirable and high-standard final products.

In the past, quality assessment of the malts was performed through a visual inspection of the color and defects of kernels; smelling was used to detect off or mold-like aromas, and chewing was used to determine flavor [6]. Currently, several analytical tests are recommended by the European Brewery Convention (EBC), American Society of Brewing Chemists (ASBC), and Mitteleuropäische Brautechnische Analysenkom (MEBAK) for a deeper physical and chemical examination [1].

The analytical techniques involved in routine practice include chromatographic, electrophoretic, fluorometric, and spectrophotometric methods, which are well recognized as expensive, time-consuming, and laborious. The tedious and complex sample preparation process of these methods, which requires the use of hazardous organic solvents, affects customer needs being met, since the significant workload of the laboratories involved in routine practice leads to delays in analysis.

Nowadays, novel analytical strategies based on green and fast alternatives to the traditional techniques have been proposed. Near-infrared spectroscopy (NIRs) is well known in the brewing industry as a reference method for moisture and total nitrogen determination in barley [8,9,10,11] and malts as well as alcohol in beer [12].

Several research works were have on the possibility of using NIR spectroscopy for the reliable and accurate estimation of other quality features of malts such as fermentability, soluble proteins, extract at 45 °C, Kolback index, friability, free-amine nitrogen (FAN) total soluble nitrogen (TSN), diastatic power (DP), and β-glucan [13,14,15,16,17]. NIRs is able to provide fast and reliable results with high levels of precision and accuracy, and the provided analytical response reflects the real composition of the matrix as no sample pre-treatment is required. In addition, the literature demonstrates NIRs’s ability in several fields to completely characterize samples of a different nature, such as foods [18,19], pharmaceuticals [20,21,22], biological fluids [23,24], substances of abuse [25,26], vegetable materials [27], agricultural products [28], and cultural heritage [29]. The coupling of NIRs with chemometrics as well as the miniaturization of instruments and equipment have led to increased interest in this spectroscopic technique due to the possibility of performing analysis on-site and ensuring real-time results [30,31,32].

Multivariate statistical data analysis permits the interpretation of a large amount of complex multidimensional data. Among the chemometrics techniques, partial least squares-discriminant analysis (PLS-DA), linear discriminant analysis (LDA)m and Soft Independent Model of Class Analogy (SIMCA) are the most known used for solving classification problems [33].

In this work, the performance of an ultracompact device operating in the NIR region of the spectrum, a microNIR spectrophotometer, in the complete investigation of malts was evaluated. An analytical procedure for the rapid and accurate identification of brewing malts resulting from different malting conditions was optimized. Specifically, a prediction model able to differentiate specialty and base malts according to differences in their chemical profile was validated. The aim was to provide a click-on analytical test as robust as possible to the variability related to sample appearance and composition as well as to origins and providers, and satisfactory results were obtained, leading to the optimization of an innovative analytical platform.

## 2. Materials and Methods

### 2.1. Samples

Twenty malt samples were included in this study. Eighteen base malts (Munich, Vienna, Pilsner and Pale Ale Maris Otter) and two specialty malts (caramel and Crystal) were purchased from Birramia.it—Enterprise SRL (Querceta, Lucca, Italy) and mr-malt.it—PAB SRL (Pasian di Prat, Udine, Italy). A detailed list of the investigated samples and their specifications are reported in Table 1. Malt grains were ground and sieved prior to spectroscopic analysis.

### 2.2. Spectroscopic Analysis

A MicroNIR OnSite W 1700 spectrophotometer (Viavi Solution Inc., JDSU Corporation, Milpitas, CA, USA) was used to collect spectra of raw materials. Four aliquots per sample (about 10 g of ground and sieved malt grains) were investigated using a special accessory for glass vials. Ten measurements were carried out on each aliquot (forty spectra per sample) in order to assess the reproducibility of the spectroscopic response. A total of 125 data point were collected with a nominal spectral resolution of 6.25 nm, an integration time, of 10 ms, and a measurement time of 0.8 s (100scan) as instrumental conditions. Calibration of the spectral response was performed at the beginning of each analytical session. A spectrum of total absorbance (dark) and total reflectance (blank) was obtained by fixing a point in the room and placing the sensor on a fluoropolymer with 99% diffuse reflectance (Spectralon). The spectrophotometer was set in reflectance mode, and spectra were collected from 900 to 1700 nm.

A linear variable filter (LVF), two tungsten light bulbs as the radiation source, and a linear indium gallium arsenide (InGaAs) array detector (128 pixels) were the equipment of the ultracompact device, resulting in a weight of 250 g, a length of 194 mm, and a diameter of 47 mm. Control of the instrument took place through a low-power wireless (Bluetooth) interface, while MicroNIR Pro software (Unscrambler X 10.4) and a MicroNIR OnSite-W system (JDSU Corporation, Milpitas, CA, USA) on a laptop allowed its management. Specifically, MicroNIR Pro was used by trained users to optimize the spectroscopic method, while the MicroNIR OnSite-W system permitted the application of the developed analytical platform directly on site, bringing the laboratory to the field.

### 2.3. Multivariate Statistical Analysis

All acquired reflectance spectra were converted to absorbance measurements and exported as ASCII files for further processing using chemometrics. A multivariate statistical analysis package, VJDSU Unscrambler Lite (Camo software AS, Oslo, Norway), was used for chemical data elaboration. First, a preliminary unsupervised analysis was carried out with the aim of studying the reproducibility of the spectroscopic measurements and identifying correlations among samples belonging to the same class. NIPALS algorithm was selected to perform principal component analysis (PCA) on all spectra from the same providers in order to evaluate whether the microNIR/chemometric approach could differentiate malts according to the different malting processes and thus discriminate samples as a function of chemical profile. After confirming this ability of the microNIR/chemometric method, two robust classifications model were developed starting from the spectra acquired on all samples involved this the study. Soft Modeling Class Analogy (SIMCA) was the class modeling technique selected for classification model optimization. A total of 75% of the entire data set was considered as the training set, while the remaining 25% of the performed measurements were processed as the test set, as reported in Table 2.

Model performances was evaluated using selectivity (%), specificity (%), efficiency (%), and false positive and false negative rates (%). Figures of merits were estimated, as reported in Table 3.

## 3. Results and Discussion

Providing an easy-to-use analytical tool for the investigation of barley malt was the final goal of this study, as the control of the raw materials used in the brewing process should ensure the quality of the final product based on consumers’ expectations. To achieve this aim, an analytical protocol based on an ultracompact device operating in the NIR region of the spectrum was optimized, and a chemometric prediction model for malt identification was validated.

### 3.1. Optimization of an Analytical Protocol for Malt Grains Using MicroNIR and Chemometrics

**Figure 1:**
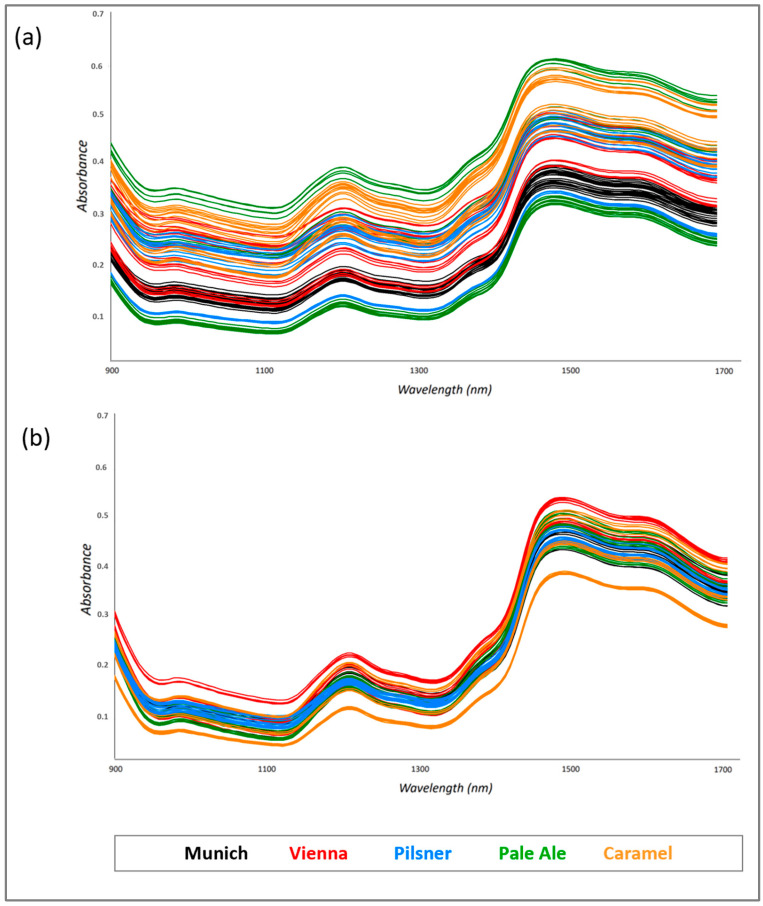
MicroNIR spectra collected from ground (**a**) and sieved (**b**) malt grains.

The first stage of this work focused on the variability in the spectroscopic signals as a consequence of the malting process and thus as a function of the different chemical compositions of the raw materials. Therefore, base and specialty malts obtained from the same provider were investigated using a MicroNIR OnSite W spectrophotometer. Figure 1 shows the measurements performed on Munich, Vienna, Pilsner, Pale Ale, and Carmel malts produced and distributed by Bestmaltz (Heidelberg, Germany).

Since NIR spectroscopy is well recognized as an analytical technique affected by the physical form of matter (i.e., particle size and thicknesses), spectra were collected from ground (Figure 1a) and sieved (Figure 1b) grains with the aim of studying the variability in the spectroscopic response as a function of the matrix features.

As expected, the spectra of the ground malt grains appeared more widely distributed along the y axis than those of sieved malt grains due to the typical undesired scatter effects of NIR radiation when samples are characterized by different particles sizes.

Table 1 shows the specifications of the products involved in this study and provided by the manufacturers. NIR spectra are certainly affected by the different characteristics of products, which depend on the malting process and sample composition. However, the dispersive effects of spectroscopic radiation do not permit the observation of the relationship among malt properties and absorption bands. For this reason, chemometrics, especially principal component analysis (PCA), was performed.

**Figure 2:**
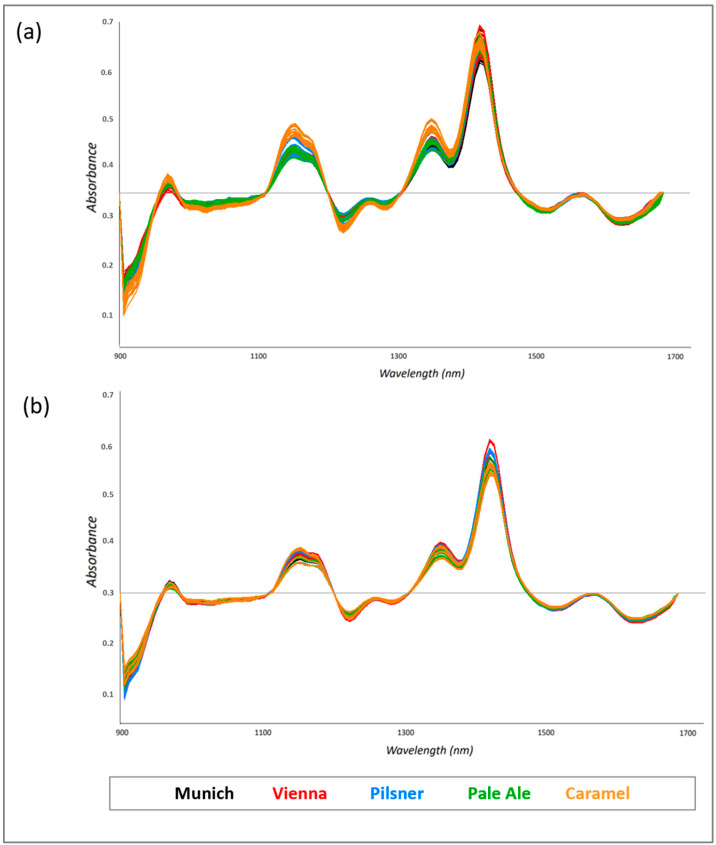
MicroNIR spectra collected from ground (**a**) and sieved (**b**) malt grains after preprocessing with first-derivative Savitzky–Golay filter.

Raw microNIR data were imported into a chemometric package, and several algorithms recommended for spectroscopic measurements were used for mathematical transformation in order to minimize scattering effects. Multiplicative scatter correction (MSC) and standard normal variate (SNV) transformation, the first- and second-order derivatives, have been proposed as the most suitable mathematical transformations for avoiding the baseline deviation caused by typical scattering phenomena, which affect reflectance measurements [35,36,37,38]. The first derivative, with Savitzky–Golay smoothing (three-datapoint window), followed by mean centering, was found to be the optimal pretreatment for spectra collected from 900 to 1700 nm, since it permitted the reduction of the additive, multiplicative and wavelength-dependent effects (Figure 2a,b).

MicroNIR analysis provided a “fingerprint” profile of malt grains that takes into account the contribution of all chemical components of the investigated matrix. The normalized spectra showed several absorption bands, mainly combined bands and overtones related to dipole changes in the bonds between hydrogen and heavier elements (e.g., oxygen or nitrogen), i.e., the typical bonds of molecules that characterize food products. Peaks at 1430 nm and 1910 nm are usually attributed to the OH absorption of water, and the bands at 1187 (CH), 1496 (NH), and 1674 (CH) nm are considered for the evaluation of protein content and nitrogen. In addition, the NIR region indicates the absorption of β-glucan and enzymes, which, for the previously mentioned parameters (water, nitrogen and protein content), are taken into account in the quality control of raw grains [11]. The contribution of a single component is difficult to identify; however, the main advantage of the spectroscopy is the possibility of obtaining a profile that includes all quality parameters of the examined samples.

The chemometric approach was found to be necessary for assessing the differences among the studied malt classes, i.e., base and specialty malts, on the basis of their quality profile.

**Figure 3:**
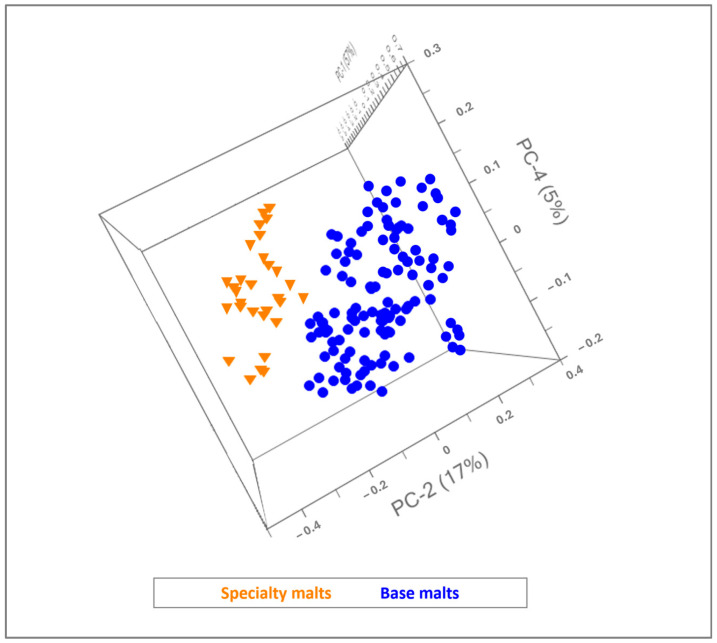
Three-dimensional scores plot resulting from PCA of base and specialty malts.

After preprocessing, a preliminary explorative study on the data set was carried out using principal component analysis (PCA) as the display method, with the aim of enhancing the relevant analytical information and pointing out correlations within the performed measurements. As demonstrated by the score plot reported in Figure 3, microNIR permitted the differentiation of the malts according to their functional properties, which depend on the malting process: in fact, base (blue) and specialty (orange) malts were found to be located in two different regions, permitting the identification of the two main classes into which the raw materials are classified.

**Figure 4:**
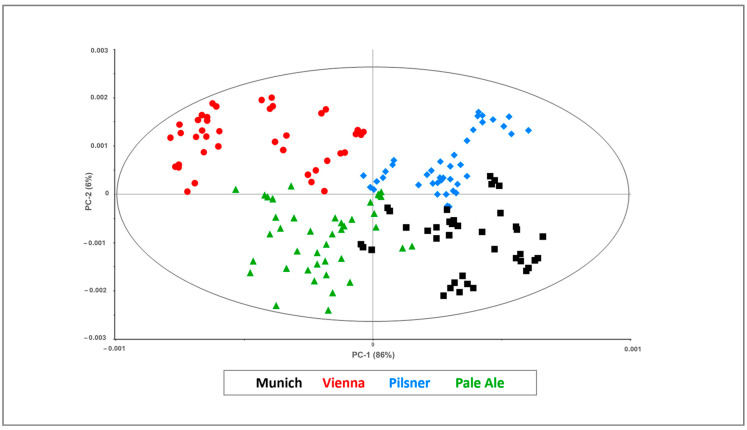
Two-dimensional scores plot resulting from base malt spectra processing by PCA.

Despite the complexity of the matrix, two clusters of samples were observed according to PC-2 (explaining 74% of the overall variance) because of differences in the expressions of the different molecules involved in the chemical profiles of specialty and base malts, which are due to different drying and roasting temperatures. Further chemometric evaluations were conducted focusing on the possibility of predicting the traditional base malt classes, which are characterized by notably differences in flavor and color profiling. The multivariate explorative analysis provided interesting outcomes, as proved by the resulting score plot in Figure 4.

A good agreement among the measurements performed on Munich (black), Vienna (red), Pilsner (green) and Pale Ale Maris Otter (light blue) was found in the space described by the first two PCs (explained 92% of the overall variance).

The results of the PCA highlighted the effectiveness of the innovative microNIR/chemometric platform in differentiating base malt variety as a function of their chemical composition; in fact, the investigated samples were found to be separated into four groups of specimens corresponding to the type of base malt. In addition, by analyzing Figure 4, it is possible to confirm that the outliers may be discarded since isolated samples, which may be recognized as doubtful, were not visualized in the scatter plot considering a 95% confidence ellipse.

### 3.2. SIMCA Model Development

The unsupervised analysis results suggested the possibility of developing and validating a predictive chemometric model for malt classification and thus providing an analytical tool for the control of product compliance. In order to ensure a prediction model that is as robust as possible, malts from nine different providers and four countries were included in this study and analyzed using the analytical protocol previously described.

A class modeling technique, Soft Modeling Class Analogy (SIMCA), was chosen for the optimization of two classification models, for separately processing the spectra from ground and sieved grains. Each malt class was modeled individually using different PCAs using the NIPALS algorithm; in all cases, validation was carried out using the cross-validation technique (leave-one-out method).

PC number, selectivity (i.e., percentage of samples correctly predicted as belonging to the studied class), specificity (i.e., percentage of samples wrongly recognized as belonging to the studied class), efficiency (i.e., the geometric average of the product within selectivity and specificity), and false positive and false negative rates were estimated by setting a class cut-off limit of 5%.

As described in Table 4, satisfactory model performance was achieved, with a good selectivity (never lower than 70.0% and 75.0% for ground and sieved malt grain models, respectively), specificity (never lower than 66.57% and 76.92% for ground and sieved malt grain models, respectively), and high efficiency (never lower than 70.71% and 73.79% for ground and sieved malt grain models, respectively). Regardless of the false positive and false negative detections, the sieved malt grain model had lower values of FPR % and FNR % than the ground malt grain model for all studied classes: Munich (2.50% vs. 4.29%), Vienna (3.41% vs. 4.29%), Pilsner (2.50% vs. 3.75%), Pale Ale (2.78% vs. 3.95%), Caramel (2.50% vs. 3.75%,) and Crystal (2.52% vs. 3.75%).

The significant outcomes proved the effectiveness of the validated models in predicting samples as belonging to one of the modeled class and thus the possibility of verifying raw material compliance for the subsequent brewing process. The possibility of analyzing samples after a minimal pretreatment was confirmed, and chemometrics effectiveness with spectra affected by dispersive effects due to the heterogeneity of sample composition was proved. This method reduces the steps in the analysis, providing a time-saving device.

The novelty of this work consists of the development of a smart analytical system that allows the real-time assessment of raw material quality that examines several parameters at the same time. The microNIR/chemometric platform enables on-site analysis thanks to the small size of the spectroscopic sensor and the possibility of importing the validated chemometric models to tablets or smartphones, providing a handheld analytical platform. The resulting device is easy to use by trained users as they perform analysis trough a single click and read results on the portable electronic system.

## 4. Conclusions

A novel analytical quality control tool based on microNIR and chemometrics for the investigation of the essential raw materials in the brewing process was proposed. A spectroscopic protocol for malt analysis was optimized using real samples. Several sources of variability were taken into account, including different beer providers from different countries as well as the physical form of the matrix. Robust chemometric models were optimized using ground and sieved barley malt grains. The models developed from sieved materials showed better performance during the prediction of the external test set, confirming the heterogeneity of the matrix and the need to reduce the particle size of grains. MicroNIR OnSite W, the latest upgrade in the portable devices available on the market, may be considered as a novelty of this study since it ensures the fast and complete characterization of specimens. The coupling with chemometrics simplifies the interpretation of the complex spectroscopic response for brewers by visualizing the classification results, i.e., the belonging of the malt to one of the modeled classes. The possibility of importing the developed chemometrics models to selected prediction software allows the obtaining of real-time results through a single click. MicroNIR requires only few seconds to acquire one spectrum, which is immediately processed with the validated model. The innovative analytical platform permitted us to obtain suitable results and to develop a fast, accurate, and easy-to-use method for suitable validation parameters. In conclusion, this approach may be considered as an optimal technology for performing the quality control of malts with a single-touch analysis as the device is entirely portable and the method is nondestructive.

## Figures and Tables

**Table 1:** Specification of malt samples.

		Provider	Country	Color (EBC)	Moisture (%)
Base Malts	Vienna	Bestmalz	Germany	8–10	4.9
		Durst Malz	Germany	6–9	4.0
		Simpson Malt	UK	5–10	3.5
		Crisp Malting	UK	6–10	4.5
	Munich	Bestmalz	Germany	11–20	4.9
		Durst Malz	Germany	20–25	4.0
		Rhön Malz	Germany	23–26	3.0–4.5
		Simpson Malt	UK	18–25	4.0
	Pilsner	Bestmalz	Germany	3–4.9	4.9
		Durst Malz	Germany	2.5–3.3	4.5
		Rhön Malz	Germany	2.8–3.8	4–4.5
		Dingemans	Belgium	3.5	4.5
		Italmalt	Italy	3.5–4.5	3–5
	Pale Ale	Bestmalz	Germany	5–7	4.9
		Simpson Malt	UK	4–6	3.7
		Crisp Malting	UK	5.5–7.5	3.5
		Muntons	UK	5–7	3.7
		Thomas Fawcett & Sons	UK	5–7.5	4.0
Specialty Malts	Caramel	Bestmalz	Germany	3–7	4.5
	Crystal	Simpson Malt	UK	167–190	5.0

**Table 2:** SIMCA model development.

	Number of Spectra	Calibration Set	Validation Set
Munich	160	120	40
Vienna	160	120	40
Pilsner	200	150	50
Pale Ale	200	150	50
Caramel	40	30	10
Crystal	40	30	10

**Table 3:** Figures of merits for model performance assessment [34].

	Equation
**Selectivity (%)**	$\frac{TP}{TP+FN}\times 100$
**Specificity (%)**	$\frac{TN}{TN+FP}\times 100$
**Efficiency (%)**	$\sqrt{Selectivity\times Specificity}$
**False positive rate (%)**	$\frac{FP}{FP+TN}\times 100$
**False negative rate (%)**	$\frac{FN}{FN+TP}\times 100$

*TP*: true positive; *TN*: true negative; *FP*: false positive; *FN*: false negative.

**Table 4:** Figures of merits for SIMCA classification models.

		PC Number	Selectivity (%)	Specificity (%)	Efficiency (%)	FPR (%)	FNR (%)
Ground malt grains	Munich	7	87.50	66.67	70.71	4.29	0.13
	Vienna	7	71.43	70.97	80.66	4.29	0.29
	Pilsner	7	75.00	67.57	75.04	3.75	0.25
	Pale Ale	6	70.00	72.73	76.28	3.95	0.30
	Caramel	6	100.00	100.00	100.00	3.75	0
	Crystal	5	100.00	100.00	91.29	3.75	0
Sieved malt grains	Munich	7	75.00	82.35	84.89	2.50	0.25
	Vienna	7	91.67	76.92	74.12	3.41	0.08
	Pilsner	6	83.33	88.24	81.35	2.50	0.17
	Pale Ale	7	80.00	77.78	73.79	2.78	0.20
	Caramel	7	100.00	80.00	89.44	2.50	0
	Crystal	7	83.33	100.00	100.00	2.52	0.17

PCs (principal components); FPR (false positives rate); FNR (false negative rate).

## Data Availability

The original contributions presented in the study are included in the article, further inquiries can be directed to the corresponding author.
